# Abiotic Stresses Cause Differential Regulation of Alternative Splice Forms of GATA Transcription Factor in Rice

**DOI:** 10.3389/fpls.2017.01944

**Published:** 2017-11-13

**Authors:** Priyanka Gupta, Kamlesh K. Nutan, Sneh L. Singla-Pareek, Ashwani Pareek

**Affiliations:** ^1^Stress Physiology and Molecular Biology Laboratory, School of Life Sciences, Jawaharlal Nehru University, New Delhi, India; ^2^Plant Stress Biology, International Centre for Genetic Engineering and Biotechnology, New Delhi, India

**Keywords:** rice, OsGATA, abiotic stress, gene family, ABA, alternative splice variants, transcription factor

## Abstract

The GATA gene family is one of the most conserved families of transcription factors, playing a significant role in different aspects of cellular processes, in organisms ranging from fungi to angiosperms. GATA transcription factors are DNA-binding proteins, having a class IV zinc-finger motif CX_2_CX_17−20_CX_2_C followed by a highly basic region and are known to bind a consensus sequence WGATAR. In plants, GATAs are known to be involved in light-dependent gene regulation and nitrate assimilation. However, a comprehensive analysis of these GATA gene members has not yet been highlighted in rice when subjected to environmental stresses. In this study, we present an overview of the GATA gene family in rice (*OsGATA*) in terms of, their chromosomal distribution, domain architecture, and phylogeny. Our study has revealed the presence of 28 genes, encoding 35 putative GATA transcription factors belonging to seven subfamilies in the rice genome. Transcript abundance analysis in contrasting genotypes of rice—IR64 (salt sensitive) and Pokkali (salt tolerant), for individual GATA members indicated their differential expression in response to various abiotic stresses such as salinity, drought, and exogenous ABA. One of the members of subfamily VII—*OsGATA23a*, emerged as a multi-stress responsive transcription factor giving elevated expression levels in response to salinity and drought. ABA also induces expression of *OsGATA23a* by 35 and 55-folds in IR64 and Pokkali respectively. However, *OsGATA23b*, an alternative splice variant of *OsGATA23* did not respond to above-mentioned stresses. Developmental regulation of the *OsGATA* genes based on a publicly available microarray database showed distinct expression patterns for most of the GATA members throughout different stages of rice development. Altogether, our results suggest inherent roles of diverse OsGATA factors in abiotic stress signaling and also throw some light on the tight regulation of the spliced variants of *OsGATA* genes in response to different environmental conditions.

## Introduction

Genetic material present in all living system as DNA eventually encodes and governs almost all the fundamental processes in live forms. Selective upregulation and downregulation of the set of genes encoded by DNA, allows an organism to respond to distinct stimuli. Transcription factors (TFs) act as synchronizing elements between action (stimuli) and reaction (gene expression). Plants being sessile, require more efficiently regulated gene expression to cope with the plethora of environmental stresses. TFs are one such key regulators governing gene expression by specifically binding to the promoter/enhancer sequences of the gene. TFs can be grouped into different categories based on the ability to bind the *cis*-acting elements in the promoter region. These transcription factors have so far been named as MADS, WRKY, MYB, bZIP (basic leucine zipper), PHD (plant homeodomain), zinc-finger, NAC (NAM, ATAF1/2, and CUC1/2), and AP2/EREBP (Apetala2/ethylene responsive element binding protein), depending upon the presence of DNA binding motifs (Shore and Sharrocks, 1995; Krishna et al., 2003; Mizoi et al., 2012; Alves et al., 2013; Ambawat et al., 2013; Sun et al., 2013). Some of these TFs are specific only to plants viz. NAC and AP2/EREBP (Mizoi et al., 2012; Shao et al., 2015). Extensive studies have been carried out to understand the role of these transcription factors in biotic and abiotic stress as well as in crosstalk between these stresses in several crop plants (Cheong et al., 2002; Pandey and Somssich, 2009; Chen et al., 2010; Lindemose et al., 2013; Gupta et al., 2016; Nutan et al., 2017).

Based on the conserved domain structure, members of the zinc-finger TFs are further classified into different families (Takatsuji, 1998). GATA transcription factors, as the name suggest, are characterized by their ability to bind W-G-A-T-A-R (W = T/A, R = G/A) sequence in the promoter region (Merika and Orkin, 1993). These are type-IV zinc-finger motif with the consensus CX_2_CX_17−20_CX_2_C sequence followed by a basic region facilitating DNA binding. GATA zinc-finger with 17–18 residues in the binding loop is a characteristic feature of animal and fungal GATA TFs. While, the plant GATA factors possess 17–20 residues in the zinc-finger loop (Reyes et al., 2004; Behringer and Schwechheimer, 2015). The DNA binding domain of these GATA transcription factors have been well-studied by NMR structures in chicken GATA1 and AreA zinc-finger protein of *Aspergillus nidulans* (Omichinski et al., 1993; Starich et al., 1998). Based on their studies, it is now known that the interaction between zinc-finger loop and specific DNA element is facilitated by hydrophobic interactions with the nitrogenous bases present in the major groove of the DNA.

The role of these GATA transcription factors have been widely studied in fungi and animals (Tsai et al., 1994; Marzluf, 1997; Scazzocchio, 2000; Tong et al., 2000; Marzluf, 2004; Pikkarainen et al., 2004). First identified in chicken, the GATA TFs have been reported to be involved in haematopoiesis (Omichinski et al., 1993). Apart from their active involvement in cell differentiation, GATA TFs are also documented to be involved in regulation of various stress signaling and metabolic pathways (Crespo et al., 2001; Xu and Kim, 2012). Fungal GATA TFs are the combination of both plant and animal GATA transcription factors in terms of the amino acid residues present in the zinc-finger loop. Diverse roles governed by GATA transcription factors in fungal cells include controlling nitrogen metabolism, circadian regulation, and siderophore production (Teakle and Kay, 1995; Arguello-Astorga and Herrera-Estrella, 1998; Haas et al., 1999; García-Salcedo et al., 2006; Chi et al., 2013).

In plants, GATA TFs have not yet been studied extensively and the knowledge about this class of transcription factors remains elusive. The first plant GATA transcription factor NTL1 identified from tobacco is a homolog of NIT-2 from *Neurospora crassa* that functions in nitrogen metabolism (Daniel-Vedele and Caboche, 1993). Recent studies carried out in other plants have revealed the involvement of GATA transcription factors in regulation of various stress responsive genes, nitrogen metabolism, flowering, developmental related genes, and in hormone signaling such as GA, auxin, and cytokinin (Richter et al., 2010, 2013; Chiang et al., 2012; Hudson et al., 2013; Behringer et al., 2014; Behringer and Schwechheimer, 2015; Zhang et al., 2015). Furthermore, active involvement of *Arabidopsis* GATA TFs in the prevention of photooxidative damage via tetrapyrrole biosynthesis (TPB) has been documented very well (Kobayashi and Masuda, 2016). *Arabidopsis* class-B GATA TFs with C-terminal LLM domain, have been widely studied and characterized as regulator of vegetative growth and development (Behringer et al., 2014). In an interesting finding by Kobayashi et al. (2017), it has been observed that *GNC-LIKE* (GNL-class B GATA) TF functioned downstream to type B ARRs in *Arabidopsis* and hence interplayed at the junction of auxin and cytokinin signaling.

With the progression of genomic tools and availability of huge genomic data, studies related to whole genome mining have become more precise and informative. In rice, 28 gene loci encoding GATA proteins have been reported more than a decade ago (Reyes et al., 2004). Since the database is evolving continuously and with the availability of refined tools, we have carried out the search for GATA family members again to identify the new proteins. In the present work, we report 28 rice loci encoding 35 putative GATA transcription factors. Taking alternative splice variants into consideration along with gene structure, number and position of GATA domain as well as the presence of accessory domains other than GATA, we have categorized them into seven subfamilies. Further, a detailed analysis of the conserved GATA domain, sequence similarity between the genes, and the phylogenetic relationship has been performed. To investigate the role of GATA transcription factors in abiotic stress signaling, we have carried out expression analysis of *OsGATA* genes in two contrasting rice genotypes i.e., salt sensitive variety—IR64 and salt tolerant landrace—Pokkali. Transcript abundant analysis suggests the differential expression of alternative splice products of *OsGATA* genes under environmental signals. Taken together, our results may open a new path to explore the potential role of GATA transcription factors in abiotic stresses in model crop plant rice.

## Materials and methods

### Characterization and nomenclature of the GATA gene family in rice

To identify all the putative GATA gene members of rice, MSU rice genome annotation project (RGAP) release 7 database (http://rice.plantbiology.msu.edu/) was scanned with GATA pfam (http://pfam.xfam.org/) domain ID PF00320. List of genes retrieved from MSU rice genome database was further confirmed by BLASTP (protein BLAST) in three different databases: NCBI (https://blast.ncbi.nlm.nih.gov/), phytozyme *V*.11 (https://phytozome.jgi.doe.gov/pz/portal.html), and plant genome database release 187 (http://www.plantgdb.org/), using *Arabidopsis* GATA1 protein sequence as the reference. Redundant sequences were omitted manually. Functional domains in the full-length protein sequence were identified using pfam, Interpro (https://www.ebi.ac.uk/interpro/), and SMART (http://smart.embl-heidelberg.de/) databases. To avoid ambiguity because of multiple names, nomenclature of these GATA genes was retained same as that of Reyes et al. (2004). However, newly identified alternative spliced products were denoted as gene number extended with suffix “a” and “b” as suggested earlier by Pareek et al. (2006).

### Chromosomal distribution of the GATA gene members

For locating the GATA members on rice chromosomes, CDS coordinates were retrieved from MSU RGAP database version 7 for each GATA gene and were placed on each of the rice chromosomes according to the physical location of the gene. Plant genome duplication database (http://chibba.agtec.uga.edu/) was used to search for the duplication events (segmental duplication and tandem duplication) of the *OsGATA* genes. Duplicated genes have been connected by dotted lines.

### Multiple sequence alignment and phylogenetic tree construction

Multiple sequence alignment of protein sequences of only GATA domain was performed using ClustalW program available in MEGA 7. The Neighbor-Joining tree was generated based on the MUSCLE alignment of the full-length OsGATA protein sequences using Jones-Taylor-Thornton (JTT) model under default setting in the MEGA 7 program. To infer phylogeny, 1,000 bootstrap replicates were taken. Gene structure display server version 2.0 (http://gsds.cbi.pku.edu.cn) was used to analyze the gene structure and for calculating the number of exon and intron in the gene sequence. The *cis*-acting elements in the promoter region of *OsGATA* genes were deduced using PlantPan version 2 (http://plantpan2.itps.ncku.edu.tw/).

### Plant material and stress treatment

Seeds of *Oryza sativa* L. cv IR64 and landrace Pokkali were rinsed thoroughly in sterile water, germinated in hydroponic set-up and raised for 7 days on half-strength Yoshida medium, at 28 ± 2°C for 12 h light and dark cycles in a plant growth chamber. For stress treatment, 7 days old seedlings were transferred to half-strength Yoshida medium supplemented with either 200 mM NaCl (for salinity), 20% PEG (for drought) or 100 μM ABA and shoot samples were harvested after 4 and 24 h of stress application. Un-treated seedlings growing in half-strength Yoshida medium were taken as control. After harvesting, samples were frozen immediately in liquid nitrogen and stored at −80°C until further use.

### Extraction of total RNA

Total RNA was isolated from the shoots of seedlings using TRIzol Reagent (Life Technologies, USA). For extraction, 100 mg tissue was homogenized to a fine powder with liquid nitrogen using pre-chilled mortar and pestle. RNA was extracted as described earlier by Soda et al. (2013). Purity and integrity of the total RNA was analyzed using spectrophotometry (Thermo Scientific, USA) and denaturing agarose gel electrophoresis respectively. The quality of RNA was checked by A_260_/A_280_ ratio and samples having A_260_/A_280_ > 1.8 were used for further analysis.

### Synthesis of first strand cDNA

First-strand cDNA was synthesized using first strand cDNA synthesis kit (Fermentas Life Sciences, USA) as per manufacturer's instructions. For removing genomic DNA contamination, DNAse (Epicenter, USA) treatment was done before proceeding for the cDNA synthesis as described earlier (Soda et al., 2013).

### Primer designing and quality check

Primers for *OsGATA* genes were designed from the region corresponding to the junction of unique 3′ UTR region and CDS sequence, using Primer Express 3.0 software (Applied Biosystems, USA). In the case of alternative splice variants, primers were designed from a unique region within the CDS sequence. The uniqueness of each primer pair to amplify a selected gene was confirmed by BLASTN using the RGAP database and NCBI databases.

### Real-time quantitative PCR analysis

The qRT-PCR analysis was performed with a Sequence Detection System ABI Prism 7500 (Applied Biosystems, USA). Reactions (final volume, 10 μl) were set up with the 2X SYBR Green PCR Master Mix (Applied Biosystems, USA), 3 μl cDNA sample and 0.5 mM of gene-specific forward and reverse primers. All the PCR reactions were performed under the following conditions: 2 min at 50°C, 10 min at 95°C and 40 cycles of 15 s at 95°C, 1 min at 58–62°C (annealing temperature range for different genes) and 30 s at 72°C in 96-well optical reaction plates (Applied Biosystems, USA). The specificity of the amplification was tested by dissociation curve analysis. Three technical replicates were analyzed for each sample and the data analysis was performed using SDS 1.4 software (Applied Biosystems, USA). For data normalization, the rice eukaryotic elongation factor 1 alpha (eEF-1α) was taken as internal control. Transcript abundance of the selected group of genes was analyzed by qRT-PCR using the 2^−ddCT^ and 2^−dCT^ method for the calculation of fold change and transcript abundance respectively (Livak and Schmittgen, 2001).

### Analysis of the transcript abundance of the *OsGATA* genes at different developmental stages of rice

To analyze the expression of the *OsGATA* genes at different developmental stages of rice, publicly available microarray database (https://www.genevestigator.com/gv/) was scanned with locus ID listed in Table 1. Expressions of these GATA genes were analyzed at germination, seedling, tillering, stem elongation, booting, heading, flowering, milking, and dough stages of rice plant development.

**Table 1:** List of the *OsGATA* genes: chromosome no; locus ID of the genes as annotated in RGAP version 7; nomenclature of the putative GATA transcripts where genes are named according to Reyes et al. (2004) and a and b denotes the alternative splice products of the same gene; subfamily of the GATA gene member; domain composition of the putative GATA transcription factor as deduced by SMART and pfam databases; start and end of the CDS coordinates on the respective chromosome; orientation of the promoter; number of exons in the gene structure; number of introns in the gene; amino acid length; predicted pI and molecular weight of the OsGATA proteins.

**Chr**	**Locus ID**	**Nomenclature**	**Subfamily**	**Domains**	**Gene start**	**Gene end**	**Strand**	**Exons**	**Introns**	**Amino acid length**	**Predicted pI**	**Molecularweight (kDa)**
Chr1	LOC_Os01g24070.1	OsGATA8a	II	GATA, LLM	13570607	13573322	+	2	1	131	9.4	14.13801
Chr1	LOC_Os01g24070.2	OsGATA8b	II	GATA, LLM	13570660	13573125	+	3	2	101	9.57	10.98943
Chr1	LOC_Os01g74540.1	OsGATA10	II	GATA, LLM	43172094	43173178	−	2	1	142	9.45	15.56477
Chr1	LOC_Os01g47360.1	OsGATA9	II	GATA, HAN	27055960	27056849	−	2	1	242	6.63	24.92279
Chr1	LOC_Os01g54210.1	OsGATA1	I	GATA	31180196	31182456	−	2	1	387	6.16	40.61048
Chr2	LOC_Os02g05510.1	OsGATA17a	VI	TIFY, CCT, GATA	2664370	2668170	+	7	6	328	5.01	34.85652
Chr2	LOC_Os02g05510.3	OsGATA17b	VI	TIFY, CCT, GATA	2664370	2668170	+	6	5	304	4.99	32.38363
Chr2	LOC_Os02g43150.1	OsGATA2a	I	GATA	25996914	25999176	+	3	2	431	8.67	45.06084
Chr2	LOC_Os02g43150.2	OsGATA2b	I	GATA	25996914	25999176	+	4	3	424	8.33	44.1476
Chr2	LOC_Os02g12790.1	OsGATA11	II	GATA, LLM	6705604	6707734	−	3	2	353	9.39	37.63795
Chr2	LOC_Os02g56250.1	OsGATA3	I	GATA	34408089	34413407	+	2	1	418	5.53	43.83134
Chr3	LOC_Os03g05160.1	OsGATA4	I	GATA	2506064	2507173	+	2	1	219	9.04	23.36916
Chr3	LOC_Os03g52450.1	OsGATA19a	VI	TIFY, CCT, GATA	30094292	30099092	−	8	7	271	8.86	24.35872
Chr3	LOC_Os03g52450.2	OsGATA19b	VI	GATA,CCT	30094292	30099080	−	7	6	223	6.14	29.02771
Chr3	LOC_Os03g47970.1	OsGATA18a	VI	TIFY, CCT, GATA	27269705	27274117	−	8	7	319	4.56	34.1237
Chr3	LOC_Os03g47970.2	OsGATA18b	VI	TIFY, CCT, GATA	27271016	27274117	−	5	4	257	4.58	27.9051
Chr3	LOC_Os03g03850.1	OsGATA27	III	GATA	1733298	1734113	−	1	0	271	6.39	28.96747
Chr3	LOC_Os03g61570.2	OsGATA12	II	GATA, LLM	34913404	34914861	+	3	2	136	9.87	15.09632
Chr3	LOC_Os03g08370.1	OsGATA22	VII	GATA, FAR1, MULE, SWIM	4272870	4277474	+	5	4	732	7.56	83.39103
Chr4	LOC_Os04g45650.2	OsGATA5	I	GATA	27000814	27003617	+	2	1	376	8.02	40.00403
Chr4	LOC_Os04g46020.1	OsGATA21a	IV	GATA	27260209	27264560	−	6	5	450	7.17	49.3862
Chr4	LOC_Os04g46020.2	OsGATA21b	IV	GATA	27260209	27264560	−	5	4	362	8.03	40.42826
Chr5	LOC_Os05g44400.1	OsGATA6	I	GATA	25832469	25834622	+	2	1	386	6.32	39.44034
Chr5	LOC_Os05g06340.1	OsGATA13	II	GATA, LLM	3245204	3247503	−	5	4	225	8.75	24.6188
Chr5	LOC_Os05g50270.1	OsGATA15	II	GATA, HAN	28817605	28818871	+	2	1	279	6.74	28.29209
Chr5	LOC_Os05g49280.1	OsGATA14	II	GATA, HAN	28271822	28272850	−	2	1	250	8.51	25.60681
Chr6	LOC_Os06g37450.1	OsGATA16	II	GATA, LLM	22155053	22157061	+	3	2	390	9.42	41.08366
Chr6	LOC_Os06g48534.1	OsGATA20	VI	TIFY, CCT, GATA	29365981	29370366	−	6	6	292	4.96	31.5719
Chr7	LOC_Os07g42400.1	OsGATA23a	VII	GATA, FAR1, MULE, SWIM	25371994	25376165	+	5	4	742	7.98	84.4357
Chr7	LOC_Os07g42400.2	OsGATA23b	VII	GATA, FAR1, MULE, SWIM	25371994	25376165	+	4	4	732	8.17	83.18586
Chr10	LOC_Os10g40810.1	OsGATA7	I	GATA	21943291	21945186	+	2	1	387	7.4	39.62075
Chr10	LOC_Os10g32070.1	OsGATA24	V	GATA, GATA, GATA	16847405	16849151	−	3	2	528	8.09	57.3008
Chr11	LOC_Os11g08410.1	OsGATA28	III	GATA	4432776	4434071	−	1	0	431	6.76	44.55654
Chr12	LOC_Os12g42970.1	OsGATA25	I	GATA	26693519	26696516	+	2	1	309	7.54	34.8304
Chr12	LOC_Os12g07120.1	OsGATA26	V	GATA, GATA	3496286	3497437	+	1	0	383	10.03	40.2071

## Results

### Members of the GATA family show huge diversity in their size, gene structure, and isoelectric point (pI)

BLASTP search in NCBI using full-length protein sequence from *Arabidopsis* GATA1 as query identified 35 sequences which contain at least one GATA zinc-finger domain (Table 1). Further, MSU RGAP database version 7 was scanned for putative numbers of GATA genes using GATA domain ID PF00320 retrieved from pfam. Domain search also yielded 35 putative GATA transcription factors encoded by 28 gene loci. Our analysis yielded additional 7 *OsGATA* transcripts which were not reported earlier. Protein BLAST searches in RGAP database also yielded similar results. These 28 GATA genes were named as *OsGATA1*-*OsGATA28* as described earlier by Reyes et al. (2004) (Table 1). The alternative spliced forms were named as “a” and “b” along with the GATA gene number (Table 1).

All the 35 GATA proteins contain at least one conserved GATA domain with a typical CX_2_CX_18−20_CX_2_ zinc-finger motif except OsGATA8b which has partially truncated zinc-finger loop. Protein domain analysis using pfam, SMART, INTERPRO databases confirmed that two of the *GATA* genes have more than one GATA domains in the encoded protein sequences. OsGATA26 has two, and OsGATA24 has three and one truncated zinc-finger loop in their encoded proteins. Protein sequences encoded by 15 *GATA* genes that contained accessory domains other than GATA might play additional roles in different physiological responses (Table 1). Among them, six members, OsGATA17, OsGATA18, OsGATA19, OsGATA20, OsGATA22, and OsGATA23 possess CX_2_CX_20_CX_2_ zinc-finger loop in GATA domain. While rest of the GATA members contain a CX_2_CX_18_CX_2_ type of domain structure. All the rice GATA members are listed in Table 1 along with the gene nomenclature, domain details, and amino acid length. However, we found many differences in the amino acid length as well as in exon/intron structure of GATA TFs from earlier reported information. Predicted amino acid length of OsGATA1, OsGATA2, OsGATA5, OsGATA6, OsGATA10, OsGATA13, OsGATA16, OsGATA20, OsGATA21, OsGATA22, OsGATA23, and OsGATA26 are 387, 431, 376, 386, 142, 225, 390, 292, 450, 732, 742, and 383 respectively but earlier reports by Reyes et al. (2004) showed the amino acid length as 386, 387, 390, 387, 140, 155, 348, 332, 303, 778, 786, and 415 respectively. In addition to amino acid length, we found differences in the number of exons. Predicted exon numbers in the *OsGATA5, OsGATA7, OsGATA10, OsGATA13, OsGATA16, OsGATA19, OsGATA20, OsGATA22*, and *OsGATA23* are 2, 2, 2, 5, 3, 8, 6, 5, and 5 respectively which were previously reported as 3, 3, 3, 3, 4, 9, 8, 7, and 6. This variation could be because of rapidly evolving genomic data and availability of refined annotation tools in the rice genome database version 7.

The GATA TFs vary in amino acid length from 101 to 742 with a predicted isoelectric point (pI) ranging from 4.56 to 10.03 and molecular weight ranging from 10.98 to 84.43 kDa. OsGATA8b was found to be the smallest protein having amino acid length 101 and molecular weight 10.98 kDa. On the other hand, OsGATA23a was the largest protein with an amino acid length of 742 and molecular weight of 84.43 kDa.

### Chromosomal location and phylogenetic relationships among the GATA family members reveal their random distribution in rice genome

**Figure 1:**
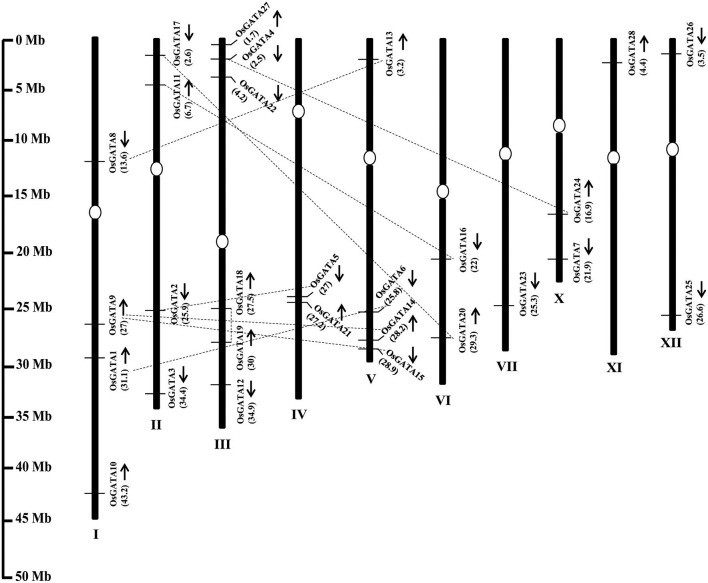
Genome architecture of the GATA gene family of rice. Graphical representation of the physical location of putative GATA transcription factors on rice chromosomes where numbers in parenthesis are the location (in megabases) of the genes at respective chromosomes. The position of centromere has been marked with an oval shape. No GATA genes were annotated at chromosome no. 8 and 9 hence not shown in the picture. Arrows marked near the gene location designates the ORF in 5′ to 3′ direction. Duplicated GATA genes on the different chromosome as well as on the same chromosome are connected by dotted lines. The scale on the left side is in megabases.

The *OsGATA* family members are randomly distributed on all the rice chromosomes, except VIII and IX (Figure 1). Maximum GATA genes i.e., six have been found to be present on chromosome III. On the other hand, only one each GATA gene has been annotated each on chromosomes VII and XI. The number of GATA genes vary from two to four on other rice chromosomes. *OsGATA5* and *OsGATA21* were clustered on chromosome IV between 27 and 27.2 Mb segments. *OsGATA14* and *OsGATA15* were present on chromosome V between 28.2 and 28.9 Mb region (Figure 1). Gene duplication has always been one of the well-known basis for the expansion of a gene family. Duplication can be either tandem; if duplicated genes are located on the same chromosome and closely linked or segmental; if duplicated genes are located on different chromosomes. We have observed eight segmental duplication events between *OsGATA* gene members (Figure 1) and one tandem duplication between *OsGATA18* and *OsGATA19* located between 27.5 and 30 Mb region of chromosome III (Figure 1).

**Figure 2:**
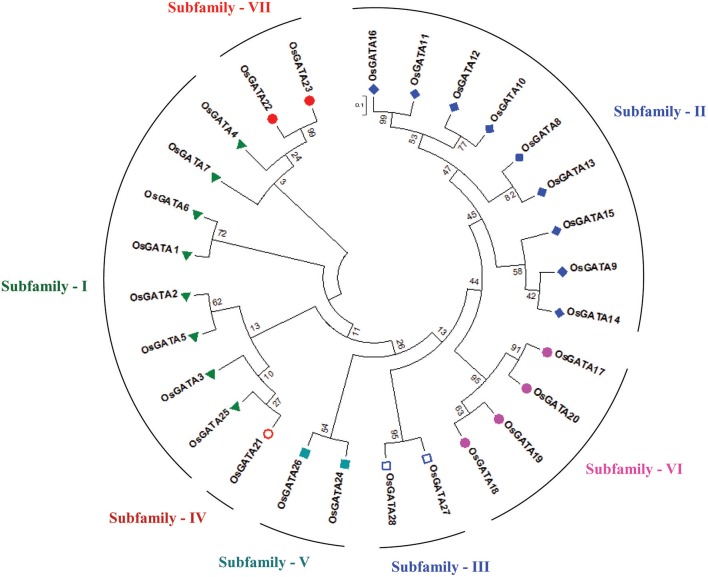
Phylogenetic tree and subfamily categorization of GATA members in rice. The Neighbor-Joining tree was constructed based on MUSCLE alignment in MEGA 7 using the Jones-Taylor-Thornton model using 1,000 bootstrap replicates under default setting. Based on the conserved domain structure in the full-length OsGATA proteins, these were grouped into 7 subfamilies. Each member belonging to one group were given similar colored shape. 0.1 scale bar corresponds to amino acid substitution rate per site.

**Figure 3:**
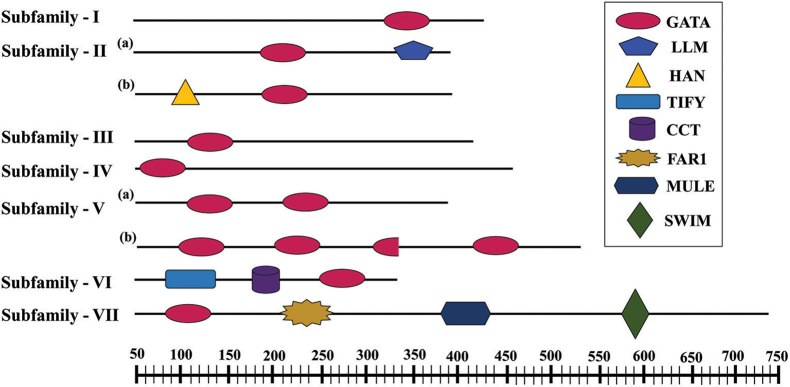
Typical domain structures of the GATA subfamily members in rice. Schematic representation of conserved domains of OsGATA subfamilies. Each domain is represented as the colored shape with the name listed at the right. The scale at the bottom of the figure represents the amino acid length of the longest OsGATA protein of particular subfamily.

**Figure 4:**
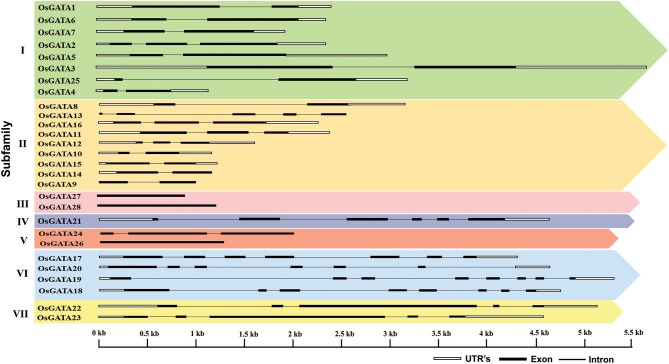
Structure of the *OsGATA* genes in the rice genome. Exon/intron structure was deduced using gene display server. Black filled boxes represents exon, black line represents introns and white boxes denotes 3′ and 5′ UTR. The scale at the bottom represents the bases in kilobase pairs.

**Figure 5:**
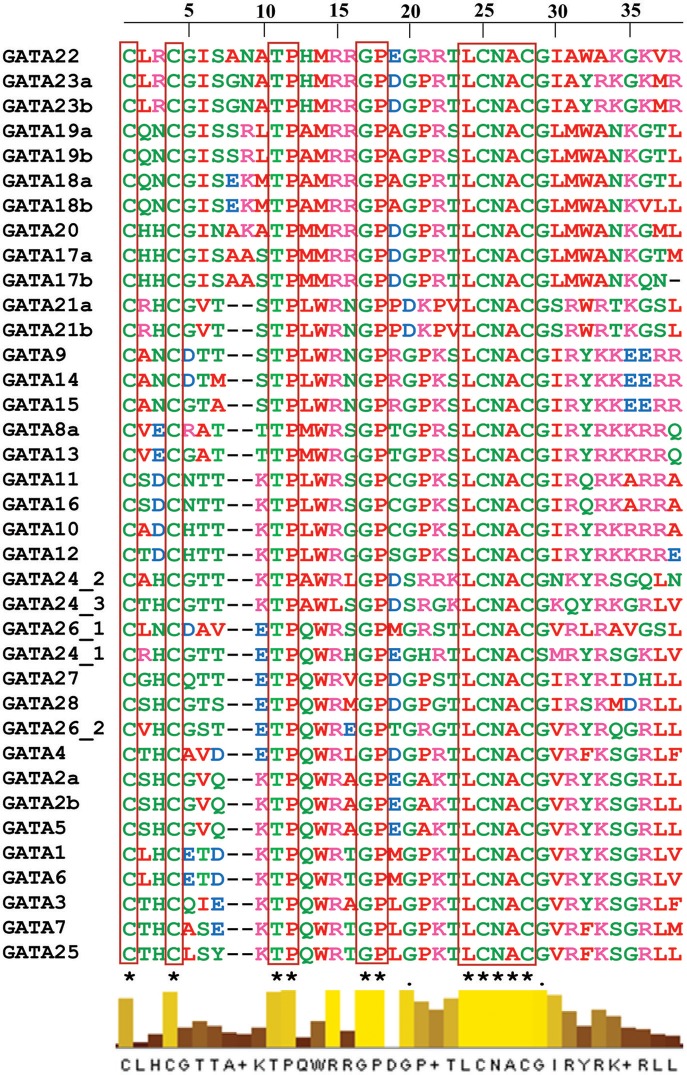
Multiple sequence alignment of the GATA domain of the OsGATA family members. Analysis of the GATA domain of 28 different *OsGATA* genes of rice showing characteristic CX_2−4_CX_18−20_CX_2_C conserved residue in the Zn-finger loop. Multiple sequence alignment was made using ClustalW program in MEGA 7. Conserved residues in all the OsGATA proteins viz. CCTPGPLCNAC are labeled with a star at bottom of the alignment. The scale at the top of the alignment denotes the amino acid length in the conserved zinc-finger loop.

Further, to compute the evolutionary distance between the genes, a Neighbour-joining tree was constructed in the Mega 7 program using the Jones Taylor Thornton (JTT) model. In this analysis, proteins with similar kind of domains got clustered in one group (Figure 2). In the course of setting up a new structural classification criteria for monocots, we have re-categorized the GATA proteins on the basis of their gene structure, the number of GATA domains, the position of GATA domain, and accessory domains (Figure 2). On the basis of homology in the GATA domain as well as the presence of accessory domain other than GATA, all the GATA genes have been subdivided into seven subfamilies (Figure 2). Typical domain structures of these TFs belonging to diverse subfamilies are presented in Figure 3. Subfamily-I has eight gene members including *OsGATA1, OsGATA2, OsGATA3, OsGATA4, OsGATA5, OsGATA6, OsGATA7*, and *OsGATA25* (Figure 2). All these GATA proteins carry a single GATA domain at the C-terminal end (Figure 3). Among them, *OsGATA1, OsGATA3, OsGATA6, OsGATA7*, and *OsGATA25* show the highest homology within the GATA domain (**Figure 5**). Subfamily-II is the largest and comprises of nine GATA genes, *OsGATA8, OsGATA9, OsGATA10, OsGATA11, OsGATA12, OsGATA13, OsGATA14, OsGATA15*, and *OsGATA16* with the GATA domain being centrally located. OsGATA8b, one of the alternative splice variant of *OsGATA8*, contains a partially truncated GATA domain. Members of the subfamily—II are complex in terms of their domain architecture (Figure 3) and have been functionally sub-categorized as B-class of GATA transcription factors in rice and *Arabidopsis* (Behringer et al., 2014; Behringer and Schwechheimer, 2015). OsGATA9, OsGATA14, and OsGATA15 possess a HAN (HANABA TARANU) domain at the N-terminal of the protein (Figure 3). On the other hand OsGATA8, OsGATA10, OsGATA11, OsGATA12, OsGATA13, and OsGATA16 possess a highly conserved LLM (leucine-leucine-methionine) domain at the C-terminal of the protein (Figure 3). Members of the subfamily-III include *OsGATA27* and *OsGATA28* (Figure 2) which contain an N-terminal located GATA domain. Though they possess similar domain structure but differ in their gene structure (Figure 4). These are intronless genes (Figure 4). The lone member of subfamily-IV, OsGATA21 came out as an outlier with an extreme N-terminal GATA domain (Figure 3). *OsGATA21* possesses six exons and five introns (Figure 4). The two members of subfamily-V, OsGATA26 and OsGATA24 contain unique 2 and 3 & 1 truncated GATA domains respectively, in the encoded protein. Subfamily-VI comprises of GATA genes which encode for GATA protein having GATA domain along with two accessory domains namely TIFY and CCT (Figure 2). *OsGATA17, OsGATA18, OsGATA19*, and *OsGATA20* belong to subfamily-VI. The alternative splice variants of the genes *OsGATA17* (a and b), *OsGATA18* (a and b), and *OsGATA19* (a and b) possess typical GATA domain with CX_2_CX_20_CX_2_ zinc-finger loop. Gene structure of the members of this subfamily is complex, having 6–7 exons in the coding sequence (Figure 4). The GATA subfamily-VII comprises of only two members, *OsGATA22* and *OsGATA23* (Figure 2). The coding sequence for these genes are interrupted by four introns and hence possess five exons (Figure 4). Typical domain structure of this subfamily includes GATA, FAR1, MULE, and SWIM domain (Figure 3). Both FAR1 (FAR Red Impaired Response1) and MULE (Mutator-like transposases) domains show sequence homology and possess C_2_H_2_ zinc-finger-like motif and SWIM domain (found in SWI2/SNF and MuDR transposases). Further, to analyze the conserved amino acid residues in the GATA zinc-finger loop; we have carried out multiple sequence alignment of the GATA domains from all the peptide sequences (Figure 5). In the case of OsGATA26, both the GATA domains were kept in analysis and numbered as OsGATA26_1 and OsGATA26_2. Similarly, all the three GATA domains of OsGATA24; OsGATA24_1, OsGATA24_2, and OsGATA24_3 were aligned along with the other GATA domains. Partially truncated GATA domains of OsGATA8b and OsGATA24_4 were not included in this analysis. Close inspection of the aligned protein sequences revealed that apart from the conserved Cys residues at Cys-1, Cys-4, Cys-25, and Cys-28 in the zinc-finger loop, few amino acid residues in between the Cys-4 and Cys-28 are also conserved. The residues Thr-11, Pro-12, Gly-17, Pro-18, Lys-24, Asn-26, and Ala-27 (Figure 5), contribute to the formation of α-helix in the zinc-finger loop; suggesting their role in maintaining the structural integrity of the domain.

### The *OsGATA* family members are differentially regulated in contrasting rice genotypes in response to salinity, drought, and ABA

**Figure 6:**
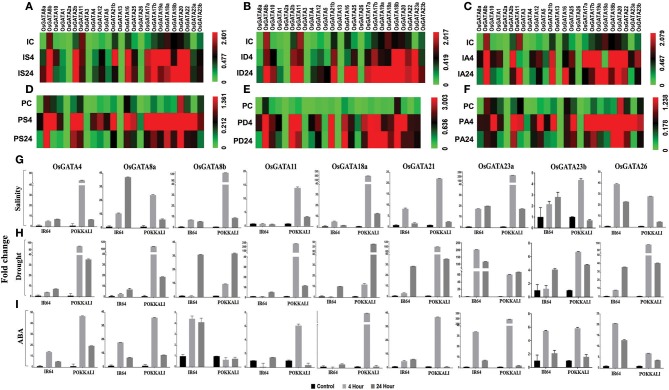
qRT-PCR based transcript profiling of the *OsGATA* genes in shoot sample of contrasting rice genotype IR64 and Pokkali in response to salinity, drought, and ABA. Heat maps showing the transcript abundance (2^−dCt^) of *OsGATA* genes in salt sensitive and salt tolerant rice genotypes, IR64 and Pokkali respectively. Expression of *OsGATA* genes in IR64 in response to **(A)** salinity, **(B)** drought, and **(C)** exogenous application of ABA. Transcript abundance of *OsGATA* family members in Pokkali in response to **(D)** salinity, **(E)** drought, and **(F)** exogenous application of ABA. Color scale at the right side of each heat map represents expression values where green is for lowest value, black for medium expression and red for highest expression. IC, IR64 Control; IS, IR64 salinity; ID, IR64 drought; IA, IR64 ABA; PC, Pokkali control; PS, Pokkali Salinity; PD, Pokkali drought; PA, Pokkali ABA. Numbers 4 and 24 corresponds to the duration of stress i.e., 4 and 24 h respectively in each treatment. **(G–I)** Histogram depicting variation in expression pattern in terms of fold change (2^−ddCt^) with respect to IR64 and Pokkali control. Fold change under **(G)** salinity stress, **(H)** drought stress, and **(I)** exogenous ABA are calculated for 4 and 24 h of stress duration with respect to control sample of each genotype and represented in the form of a histogram. Data shown are mean of three replicates ±*SD* of three replicates.

To comment on the possible roles of *OsGATA* family members in abiotic stress response, transcript abundance of *OsGATA* genes were analyzed in two contrasting rice genotypes, IR64 and Pokkali in response to distinct abiotic stresses such as salinity, drought, and stress responsive phytohormone ABA (Figure 6). To check the expression of all the 35 transcripts, unique primer combinations (Table S2), from the junction of 3′ UTR and CDS sequence were designed. However, *OsGATA6, OsGATA7, OsGATA9, OsGATA14, OsGATA15, OsGATA19b, OsGATA21a, OsGATA24, OsGATA27*, and *OsGATA28* could not be amplified from any of the cDNA used in this analysis; therefore these were kept out of the expression analysis. Expression data of remaining *OsGATA* genes has been presented in the form of heat map (Figures 6A–F).

The qRT-PCR analysis revealed unique findings for the OsGATA family expression. Differential accumulation of the *OsGATA* transcripts was observed in IR64 and Pokkali (Figures 6A–I). Basal level expression of *OsGATA2b, OsGATA8b, OsGATA11, OsGATA16, OsGATA17b, OsGATA20, OsGATA22, OsGATA23b*, and *OsGATA25* were comparatively higher in both the rice genotypes (Figures 6A–F). However, expression of some of the genes was found to be genotype specific. GATA members such as *OsGATA17a, OsGATA18a, OsGATA18b, OsGATA19a*, and *OsGATA21b* were highly expressed in IR64 under control conditions. On the other hand, expression of *OsGATA1* and *OsGATA10* were higher only in Pokkali genotype under the control conditions (Figures 6D–F). Also, the transcript levels of the *OsGATA2a* and *OsGATA13* in IR64 and Pokkali were not affected by any of the aforesaid stresses (Figures 6A–F).

*OsGATA3* was upregulated in response to exogenous ABA in both the genotypes (Figures 6C,F). However, induction was more pronounced in Pokkali. *OsGATA26* accumulated in response to salinity and drought in both the genotypes, but accumulation pattern varied with duration of stress given (Figures 6A,B,D,E). In Pokkali, the gene was upregulated after 4 h of salinity and drought stress. However, in IR64, under drought stress, transcripts accumulated at 24 h of stress while under salinity stress, induction is marked at 4 h (Figures 6A,B).

In IR64, members of subfamily-VI such as *OsGATA17a, OsGATA17b, OsGATA18a, OsGATA18b, OsGATA19a*, and *OsGATA20* as well as *OsGATA22*, a member of subfamily-VII, maintained higher transcript level at 24 h of all the applied stresses (Figures 6A–F). *OsGATA23a* also maintained higher transcript level at 24 h but only under drought in IR64. Contrarily, in Pokkali, the expression pattern of the gene members from subfamily VI and VII varied with the duration of stress (Figures 6D–F). Though induction was observed with the onset of stress (4 h) in response to salinity, drought as well as ABA (Figures 6D–F); but the transcripts level declined as the stress continued for 24 h, in salinity and ABA (Figures 6D,F); finally maintaining a level higher than the control. Under drought, transcript levels maintained a similar accumulation at the end of 24 h as that of 4 h level (Figure 6).

In this study, differential regulation of the alternative spliced variants of some of the *OsGATA* genes was also identified (Figures 6A–I). The transcript level of *OsGATA2a*, a spliced variant of *OsGATA2*, remained unchanged in all the applied stresses for both the rice genotypes (Figures 6A–F). Interestingly, expression of *OsGATA2b* was induced in response to salinity, drought, and ABA in both the genotypes at early stress duration (4 h). However, responses varied from one genotype to another during the later duration of stresses. In case of IR64, *OsGATA2b* showed higher transcript levels at 24 h post salinity and drought while the expression was downregulated in the ABA treated samples (Figures 6A–C). On the other hand, downregulation was seen in the Pokkali samples for salinity as well as ABA stress treatment at the end of 24 h while a higher level was seen in case of drought stress imposed samples for the same duration of time (Figures 6C–F).

Expression of *OsGATA8b*, the alternative spliced variant of the gene *OsGATA8*, was higher than the *OsGATA8a* in both the genotypes under all the three conditions tested (Figures 6A–F). Similarly, splice variants of *OsGATA23* also varied in their expression pattern in both the genotypes in response to abiotic stresses (Figures 6A–F). In IR64 as well as in Pokkali, *OsGATA23a* showed an induction at 4 h stress duration and continued to maintain higher transcript till the end of 24 h of salinity and drought but it was downregulated in response to ABA at 24 h in both the genotypes (Figures 6A–F). On the other hand, transcripts of *OsGATA23b* were relatively low as compared to the *OsGATA23a* in both the genotypes. It can be said that, though the gene showed induction at the onset of stress but ultimately its transcript levels declined as the stress prolonged for 24 h in both the genotypes (Figures 6A–F).

In terms of fold change, maximum induction i.e., 40-folds was observed for *OsGATA8a* and *OsGATA26* in IR64 under salinity stress (Figure 6G). Furthermore, in Pokkali salinity induced transcript levels of *OsGATA8b, OsGATA18a*, and *OsGATA23a* by more than 100-folds (Figure 6G). Interestingly in IR64, drought stress and ABA application lead to induction of *OsGATA23a* by more than 150- and 35-folds respectively (Figures 6H,I).

Similarly in Pokkali, it was seen that drought and ABA modulate the expression of a set of genes that included *OsGATA4, OsGATA8a*, and *OsGATA21b* upregulating them by more than 100- and 35-folds with respect to control under drought and ABA treatment respectively (Figures 6H,I). Similarly, *OsGATA11* and *OsGATA26* show more than 200- and 100-fold change respectively in transcript level under the drought stress. On the other hand, *OsGATA18a* showed ABA-dependent upregulation up to 300-folds and *OsGATA23a* showed a 250-folds change in response to ABA (Figure 6I).

The expression of the *OsGATA23a* was also found to be higher as compared to the *OsGATA23b*. In our study, *OsGATA23a* was identified as multi-stress responsive gene as it showed maximum upregulation for salinity, drought as well as ABA stress. In both the genotypes, this gene was induced at 4 h of stress. In the case of IR64, induction was 40-fold while in Pokkali more than 60-fold induction was observed. Since ABA is a stress hormone, it not only governs stomatal opening but also acts as a master regulator of abiotic stress signaling. Our data suggested that some of the *OsGATA* genes are highly responsive to ABA, hence these *OsGATA* genes might also be interplaying an important role at the junction of stress signaling cascade.

To understand the regulation of these GATA transcription factors, we analyzed the *cis*-acting elements in the promoter region of *OsGATA* genes (Table S1). Binding sites of various stress responsive transcription factors such as AP2 (Apetala2), ERF (Ethylene Response Factors), bHLH (basic helix loop helix), bZIP (basic leucine zipper), MADF (myb/SANT-like domain in Adf-1), Myb, NAC (NAM, ATAF1/2 and CUC1/2), WRKY, and MADS box were found. Interestingly, we found that promoter region of almost all the *OsGATA* genes possess GATA binding sites indicating that the expressions of *OsGATA* genes might be regulated by GATA transcription factors themselves.

### Expression of the *OsGATA* genes is developmentally regulated

**Figure 7:**
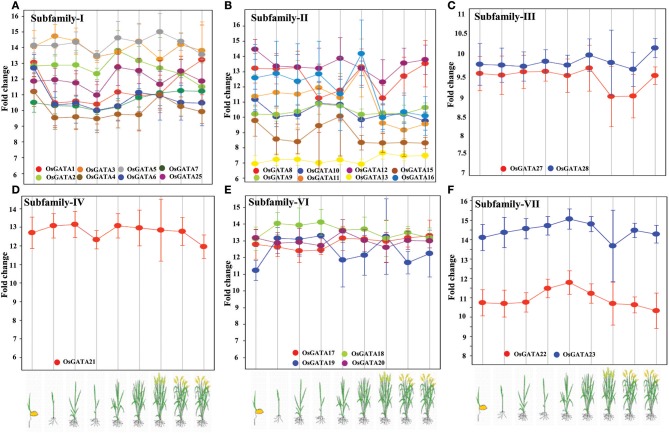
Rice GATA gene members are developmentally regulated. Expression pattern of the *OsGATA* gene **(A)** Subfamily I, **(B)** Subfamily II, **(C)** Subfamily III, **(D)** Subfamily IV, **(E)** Subfamily VI, and **(F)** Subfamily VII based on genevestigator database (https://www.genevestigator.com/gv/). No data could be retrieved for subfamily V. Rice developmental stages shown in the graphs from left to right are germination, seedling, tillering, stem elongation, booting, heading, flowering, milk, and dough. The scale at left represents the level of expression (signal intensity on Affymetrix rice genome array).

To comment on the role of OsGATA transcription factors in rice developmental processes, we analyzed the expression data from publicly available microarray database, Genevestigator (Table S3). Transcript abundance of distinct members of the *OsGATA* gene subfamilies at various developmental stages like germination, seedling, tillering, stem elongation, booting, heading, flowering, milking, and dough stage was checked (Figure 7). Interestingly, transcripts of members of the subfamily IV and VI were found to be comparatively abundant throughout all the developmental stages of the rice plant. On the other hand, members of the subfamily II showed huge variation in terms of fold change at the different stages from seedling to maturity. The members of subfamily I and II showed mixed expression pattern. Our analysis showed that the *OsGATA12* gene was induced the most during seedling stage amongst all *OsGATA* genes. Expression of *OsGATA22*, members of subfamily VII, was observed to be low as compared to other GATA gene members (Figure 7). On the other hand *OsGATA23* falls under higher expression group (Figure 7).

## Discussion

Regulated expression of a gene is essential for distinct physiological and biochemical processes in a living system. Transcription factors play a key role in governing gene regulation and exhibiting differential expressions under different physiological and environmental conditions. In this study, we present a detailed analysis of GATA transcription factors in rice. Total 28 *OsGATA* genes were identified in corroboration with the previous finding by Reyes et al. (2004). Besides, we have reported 35 putative OsGATA TFs encoded from 28 *OsGATA* genes. Newly identified alternative spliced products of the *OsGATA* genes—*OsGATA8, OsGATA12, OsGATA17, OsGATA18, OsGATA19, OsGATA21*, and *OsGATA23*, contribute for the expansion of complex rice GATA TFs family. Alternative splicing is the hallmark of the complex transcriptome in eukaryotes, as the splice variants can drive the diverse functions of a gene (Park and Graveley, 2007; Syed et al., 2012). In our analysis, we have observed that the splice variants of the *OsGATA2, OsGATA8, OsGATA18*, and *OsGATA23* respond differently to diverse environmental conditions.

Taking into account, the distinct domain architecture of the GATA proteins, their complex gene structure and phylogenetic analysis, the rice GATA genes were categorized into seven subfamilies. Subfamily II is the largest with nine GATA members viz. *OsGATA8, OsGATA9, OsGATA10, OsGATA11, OsGATA12, OsGATA13, OsGATA14, OsGATA15*, and *OsGATA16*. In this case, the GATA binding domain is centrally located and the GATA domain coding region is split into two halves by an intron sequence. However, members of the subfamily-II have been well characterized and functionally categorized into B-class of GATA gene family (Behringer and Schwechheimer, 2015). Additional domains like LLM and HAN present in the members of subfamily-II, have been well-studied and identified as functional component of plant growth in *Arabidopsis*, tomato, *Brachypodium*, and barley (Behringer et al., 2014). They are involved in regulation of various physiological as well as structural transitions in plants such as germination, hypocotyl elongation, embryo development, flower development, and senescence (Behringer et al., 2014; Behringer and Schwechheimer, 2015). These additional domains present in rice GATA TFs might also be involved in various stages of rice plant development With the eight members grouping together, subfamily I is the second largest. Members of the subfamily I possess C-terminal GATA domain with typical CX_2_CX_18_CX_2_ zinc-finger loop. Subfamily III possesses two GATA members wherein the GATA domain is located at the N-terminal. *OsGATA21* is the only member of subfamily IV with unique six exons in the gene sequence and extreme N-terminal zinc-finger loop. Subfamily V includes OsGATA26 and OsGATA24 with 2 and 3 GATA domains respectively. Members of the subfamily VI and VII are the most peculiar as they possess CX_2_CX_20_CX_2_ like zinc-finger loop for DNA binding. Apart from GATA domain, members of subfamily VI have TIFY and CCT domains. On the other hand, members of the subfamily VII contain FAR1, MULE, and SWIM domains. Previous studies related to GATA transcription factors in animals suggested that the C-terminal GATA finger proteins are involved in recognizing DNA elements in the promoter region, while N-finger GATA either assist this binding by stabilizing the DNA-protein complex or are involved in other physiological processes (Ko and Engel, 1993; Pedone et al., 1997). Like in animal system, extra GATA domain present in rice OsGATA24 and OsGATA26 might also be playing crucial roles in diverse cellular processes. Expression analysis carried out under salinity and drought stress resulted in more than 50-fold change in *OsGATA26* transcripts. Furthermore, exogenous application of ABA also upregulated expression of *OsGATA26* by more than 10-folds. These findings indicate that the additional GATA domains present in OsGATA26 might be regulating its role in abiotic stress signaling.

GATA TFs have been shown to play an integral role in light-mediated signaling (Putterill et al., 1995; Chen et al., 2009). In our genome-wide investigation, we have found that members of subfamily VI possess a unique CCT domain which is also found in TOC1 (Timing of Cab Expression 1) and CO (CONSTANS) proteins. Both of these proteins are important components of light signaling, circadian clock, and flowering (Robson et al., 2001; Más et al., 2003; Wenkel et al., 2006; Gendron et al., 2012). Our analysis also revealed the presence of FAR1 domain along with GATA domain in members of subfamily VII. The FAR1 domain is found in proteins involved in phytochrome signaling (Hudson et al., 1999; Lin et al., 2008; Li et al., 2011). Although, no clear evidence for the involvement of rice GATA TFs in light signaling has been established yet, the domain analysis presented here indicates that OsGATA22 and OsGATA23 bearing FAR1 domain might be crucial components of phytochrome signaling and the circadian clock. In higher plants, proteins with FAR1 and SWIM domains are involved in phytochrome signaling. This suggested that GATA TFs having accessory domains like FAR1, SWIM and MULE domain might play a role in light-regulated signaling in plants. Luo et al. (2010) have demonstrated that one GATA TF, GATA2 from *Arabidopsis* functions at the junction of brassinosteroid and phytochrome signaling. This indicates that GATA transcription factors with similar domain structures may be functioning via a similar pathway.

To comment on the role of these GATA TFs in abiotic stresses, we have analyzed the relative transcripts level in response to salinity, drought, and multi-stress responsive phytohormone ABA. We found that under non-stress conditions, the transcript level of some of the GATA genes was higher in salt tolerant genotype Pokkali as compared to salt sensitive variety IR64. *OsGATA1* and *OsGATA10* maintained higher transcript levels in non-stress conditions in Pokkali. Earlier, from our lab, it has been reported that salt tolerant Pokkali maintains higher constitutive level of stress related genes which are otherwise induced in salt sensitive IR64 (Karan et al., 2009; Kumari et al., 2009; Soda et al., 2013; Nutan et al., 2017). Recent study on expression of *Saltol* QTL localized transcription factors revealed that Pokkali has an abundance of these transcripts which are otherwise induced under stress conditions only in IR64 genotype (Nutan et al., 2017). Our findings also indicate that above mentioned GATA TFs, OsGATA1, and OsGATA10 might also be acting as mediators of abiotic stress signaling and response.

Furthermore, expression of some of the *OsGATA* genes are stress specific but not genotype-specific. *OsGATA3* is specifically induced in response to ABA and *OsGATA26* in response to salinity and drought in both the genotypes. On the other hand, expression of members of subfamily VI and subfamily VII were higher in all the applied stresses. These findings suggest that OsGATA proteins with accessory domains may be function via a cross talk between different abiotic stress signaling pathways. Moreover, no change in the expression of *OsGATA2a* and *OsGATA13* was observed with respect to any of the applied stresses in both the genotypes. This shows that both of these genes might not be part of abiotic stress signaling rather might be functioning in the rice developmental pathways. There are reports confirming the role of GATA TFs in distinct plant development and physiological processes (Liu et al., 2005; Lu et al., 2017).

In addition to this, differential regulation of alternative spliced forms of an *OsGATA* gene is also observed in response to abiotic stresses. *OsGATA23a*, a spliced variant of *OsGATA23* is highly expressed in all the three stresses while relative expression of *OsGATA23b* was very low under salinity, drought, and ABA. Numerous reports are available justifying the role of alternative spliced forms in different environmental circumstances (Mastrangelo et al., 2012). Recent studies carried out by Jiang et al. (2017) reveal that heat stress alters the expression of some RNA binding proteins which in turn promotes the alternative splicing in grape. Our data also shows stress specific expression of the alternative spliced variants of *OsGATA* genes suggesting a similar mechanism operating in rice for the regulation of *OsGATA* genes under abiotic stresses.

To further gain insight into the regulation of *OsGATA* genes, we looked for the various *cis*-acting elements in the promoter region of *OsGATA* genes (Table S1). Binding sites for various stress responsive TFs were studied. Interestingly, *OsGATA23* is the only gene having MADS box binding site in the promoter region. In rice, it has been reported that members of OsMADS gene family are regulators of abiotic stress signaling (Arora et al., 2007). Recently, it was reported that many of the OsMADS TFs are localized in *Saltol* QTL and are differentially regulated in contrasting rice genotypes (Nutan et al., 2017). As such it can be said that the multistress responsive nature of the OsGATA23 may be because of the binding and regulation via MADS-box TFs.

We have also examined the expression of *OsGATA* genes at different stages of rice plant development. In this analysis, we observed that members of subfamily IV and VI vary in their expression pattern from a medium to high level as the development proceeds from seedling to dough stage. In contrast, *OsGATA* gene members from subfamily I, II, and III showed varied expression level ranging from a low to high. At seedling stage, expression of *OsGATA12* was observed to be highest. In an interesting study carried out in *Arabidopsis*, it has been reported that BME3 which is a GATA transcription factor, plays a significant role in seed germination (Liu et al., 2005). Transgenic *Arabidopsis* seeds under-expressing BME3 showed delayed germination. Our analysis based on genome-wide expression analysis clearly shows that *OsGATA* genes might be playing a crucial role in rice developmental pathways. Behringer et al. (2014) have documented that mutation in LLM domain of B-GATA transcription factor of *Arabidopsis* affects plant growth and hypocotyl elongation. Besides this, distinct GATA factors are identified as functional component of shoot apical meristem (SAM) development, chloroplast development, flowering, growth, and cell division in *Arabidopsis* (Zhao et al., 2004; Chiang et al., 2012). Therefore, it is clearly evident that the GATA factors are one of the important messengers for plant cell transitions and differentiation.

Our *in silico* analysis and expression data markedly sheds some light on the complex circuitry of rice GATA transcription factors and their potential role in various physiological processes as well as in abiotic stress signaling. Additionally, it also opens a path for the future exploration and characterization of *OsGATA* genes to further understand the molecular regulatory network of transcription factors in rice.

## Conclusions

In this study, whole genome analysis of *OsGATA* gene family in rice was done to identify putative OsGATA transcription factors encoded by rice genome. Their gene, as well as protein structure, phylogeny, chromosomal location, was deduced and expression pattern under environmental stresses as well as at various rice developmental stages was analyzed. A total of 35 OsGATA TFs encoded from 28 loci were found to be randomly distributed on rice chromosomes and were categorized into seven subfamilies. Members of the *OsGATA* gene family were differentially expressed under abiotic stresses. *OsGATA23a* is multi-stress responsive as it showed high transcript levels induced by salinity, drought as well ABA treatment. Overall, the present work in aimed at providing not only an insight into the diversity of OsGATA TFs, guides toward functional cataloging of OsGATA TFs in response to environmental signals, but to establish leads for understanding the mechanism governing abiotic stress adaptability in rice via GATA TFs.

## Author contributions

PG carried out *in silico* analysis. PG and KN did transcript abundance analysis. PG and AP drafted the figures, tables, and manuscript. AP and SS-P conceived and designed the experiments. All authors read and approved the final manuscript.

### Conflict of interest statement

The authors declare that the research was conducted in the absence of any commercial or financial relationships that could be construed as a potential conflict of interest.
