# Supplementary material for: Abiotic Stresses Cause Differential Regulation of Alternative Splice Forms of GATA Transcription Factor in Rice
Source: Front Plant Sci. 2017 Nov 13;8:1944. doi: 10.3389/fpls.2017.01944 (PMC5693882; doi:10.3389/fpls.2017.01944)
Supplement: Supplementary file 1 [file Table1.DOCX]

**Table S1:** List of the cis-acting elements in the promoter region of OsGATA genes. Numbers indicate the number of binding sites of the corresponding TFs in the promoter region.

|  | **AP2** | **AP2/ERF** | **B3/ARF** | **AP2/B3/RAV** | **bHLH** | **bZIP** | **C2H2** | **GATA** | **MADF** | **Myb/SANT** | **NAC/NAM** | **WRKY** | **MADS box** | **EIN3** | **E2F** |
| --- | --- | --- | --- | --- | --- | --- | --- | --- | --- | --- | --- | --- | --- | --- | --- |
| **OsGATA8** | 3 | 2 | 0 | 1 | 3 | 8 | 4 | 4 | 0 | 7 | 0 | 0 | 0 | 1 | 0 |
| **OsGATA10** | 4 | 4 | 0 | 0 | 2 | 0 | 0 | 2 | 2 | 3 | 3 | 5 | 0 | 0 | 0 |
| **OsGATA9** | 9 | 4 | 0 | 0 | 2 | 2 | 3 | 6 | 0 | 1 | 3 | 2 | 0 | 1 | 0 |
| **OsGATA1** | 3 | 0 | 0 | 0 | 4 | 4 | 0 | 10 | 1 | 2 | 3 | 2 | 0 | 2 | 0 |
| **OsGATA2** | 0 | 0 | 0 | 0 | 3 | 3 | 2 | 3 | 1 | 5 | 5 | 4 | 0 | 0 | 0 |
| **OsGATA11** | 2 | 0 | 0 | 0 | 1 | 2 | 1 | 3 | 1 | 3 | 5 | 3 | 0 | 0 | 0 |
| **OsGATA3** | 0 | 0 | 0 | 0 | 1 | 6 | 5 | 7 | 3 | 4 | 3 | 2 | 0 | 0 | 0 |
| **OsGATA4** | 5 | 0 | 0 | 0 | 6 | 5 | 1 | 4 | 1 | 6 | 1 | 1 | 0 | 0 | 0 |
| **OsGATA27** | 0 | 0 | 0 | 7 | 1 | 2 | 0 | 3 | 3 | 6 | 0 | 0 | 0 | 0 | 0 |
| **OsGATA12** | 11 | 0 | 0 | 0 | 6 | 4 | 2 | 5 | 3 | 7 | 3 | 2 | 0 | 1 | 2 |
| **OsGATA21** | 8 | 0 | 0 | 0 | 0 | 6 | 1 | 1 | 2 | 4 | 2 | 2 | 0 | 0 | 0 |
| **OsGATA6** | 0 | 0 | 0 | 0 | 0 | 16 | 4 | 1 | 1 | 5 | 0 | 0 | 0 | 1 | 0 |
| **OsGATA13** | 0 | 0 | 0 | 0 | 2 | 5 | 4 | 9 | 0 | 7 | 1 | 0 | 0 | 2 | 0 |
| **OsGATA15** | 1 | 2 | 0 | 2 | 2 | 5 | 1 | 0 | 1 | 10 | 3 | 2 | 0 | 1 | 0 |
| **OsGATA14** | 1 | 0 | 0 | 1 | 1 | 3 | 3 | 12 | 1 | 7 | 4 | 5 | 0 | 3 | 0 |
| **OsGATA16** | 2 | 2 | 1 | 1 | 3 | 10 | 3 | 10 | 1 | 3 | 2 | 1 | 0 | 0 | 0 |
| **OsGATA7** | 0 | 1 | 0 | 1 | 4 | 4 | 2 | 2 | 4 | 4 | 3 | 0 | 0 | 2 | 0 |
| **OsGATA28** | 13 | 5 | 2 | 0 | 4 | 3 | 1 | 9 | 0 | 8 | 0 | 0 | 0 | 3 | 0 |
| **OsGATA25** | 1 | 1 | 0 | 0 | 4 | 0 | 0 | 1 | 2 | 4 | 3 | 0 | 0 | 1 | 0 |
| **OsGATA26** | 7 | 0 | 0 | 0 | 1 | 5 | 6 | 5 | 1 | 12 | 2 | 0 | 0 | 0 | 0 |
| **OsGATA24** | 6 | 3 | 0 | 2 | 0 | 2 | 3 | 3 | 0 | 4 | 0 | 0 | 0 | 1 | 0 |
| **OsGATA17** | 0 | 0 | 0 | 0 | 2 | 2 | 5 | 4 | 1 | 5 | 5 | 3 | 0 | 2 | 0 |
| **OsGATA19** | 13 | 2 | 0 | 0 | 5 | 0 | 0 | 1 | 1 | 2 | 4 | 4 | 0 | 0 | 0 |
| **OsGATA18** | 9 | 7 | 0 | 0 | 0 | 2 | 0 | 5 | 1 | 4 | 2 | 2 | 0 | 2 | 0 |
| **OsGATA20** | 16 | 6 | 0 | 0 | 3 | 1 | 1 | 2 | 1 | 5 | 1 | 0 | 0 | 2 | 0 |
| **OsGATA22** | 9 | 5 | 0 | 0 | 2 | 5 | 1 | 3 | 3 | 3 | 3 | 0 | 0 | 0 | 0 |
| **OsGATA23** | 1 | 0 | 0 | 0 | 0 | 0 | 1 | 3 | 7 | 5 | 1 | 0 | 3 | 0 | 0 |

**Table S2:** List of primers used for the detection of transcripts accumulation of various OsGATA genes under abiotic stresses.

| **LOC No** | **Name** | **FP** | **RP** |
| --- | --- | --- | --- |
| LOC_Os01g24070.1 | OsGATA8a | ATGGGCTCCACCGATCGGA | CACATGGGCGTGGTGGTGGC |
| LOC_Os01g24070.2 | OsGATA8b | TTGCTACATGGCTGACAATATCCT | TGGTGTGTGCTTTGTATGTAGTATGTACT |
| LOC_Os01g74540.1 | OsGATA10 | GGAAGAAGAAGCAGCGATTCC | GCTTCGGTGACTGCCATAGC |
| LOC_Os01g54210.1 | OsGATA1 | CATGCTGTTCGACGGAGTGT | TCCTTAGGTGGTGGTGGATCA |
| LOC_Os02g43150.1 | OsGATA2a | TCGGGCCGCCTCCTCCCGGA | CTTGCGGCGCATCTCGAGCA |
| LOC_Os02g43150.2 | OsGATA2b | CGCTACAAGTCGGGCCGCCT | GGGTCTCCTTCTTGCGGTGG |
| LOC_Os02g12790.1 | OsGATA11 | GAGGGCAACAACCAGAGTACTATGT | CAAAAGATCACAAGGCACACACA |
| LOC_Os02g56250.1 | OsGATA3 | GCGAGCCCCACCTTCAT | GCGCATCTCCACCACCTT |
| LOC_Os03g05160.1 | OsGATA4 | ACAAGAAGTGATGGAACAAAAAACC | CGCTAATCGTCTCCGTCCAA |
| LOC_Os03g61570.2 | OsGATA12 | GCGGCCATCCTCCTCAT | TGCTGTTGCTAGGTGGGTAGGT |
| LOC_Os04g45650.2 | OsGATA5 | CGCCGTGGCGTCGTT | GACTAATACGGCTGGAAAAATGTACA |
| LOC_Os04g46020.2 | OsGATA21b | TTCCAAAGCTCCGTAGGTCA | CTAAGGACGAAATAGGAAAT |
| LOC_Os05g06340.1 | OsGATA13 | CAGCCTCCAAGTGATCTCTGAA | GGCACACAATTTCCCTCCAT |
| LOC_Os06g37450.1 | OsGATA16 | CTGACGCCGCCATGCT | GCATGCAGAGATGAAGTATAGCTTTT |
| LOC_Os12g42970.1 | OsGATA25 | TGACATACACTCCAATTCTCATAAGAAG | GTTCCCGACCACCAGTTCAC |
| LOC_Os12g07120.1 | OsGATA26 | CCGCCGAGCCAATCC | GCGACTGGCACGCTTACC |
| LOC_Os02g05510.1 | OsGATA17a | TGCAATGCATGTGGACTTAT | AACATCATTGGTTGCAGCTG |
| LOC_Os02g05510.3 | OsGATA17b | GGCAAACAAGCAGAACGGAAT | GACTGCGATTCATGGCCATT |
| LOC_Os03g52450.1 | OsGATA19a | ATGGCGGCGGAGCCCCCGGC | CAGCTGCTCCGACGCCGCGC |
| LOC_Os03g47970.1 | OsGATA18a | GTACCCTGAGAAATTGCC | ACCAATTCCTTGTTGTCGAT |
| LOC_Os03g47970.2 | OsGATA18b | TGACACCGGCAATGCGCCGT | CTAGCTCATTAATACATGCA |
| LOC_Os06g48534.1 | OsGATA20 | TTCTGCATATGTTGTAAGCCATGA | CACCCAAGAGTCTAACAAGAAATAAAATT |
| LOC_Os03g08370.1 | OsGATA22 | TTCGCCTGAACCATGAAACA | TGTCTTGTGTCATTGTCTTTGGAA |
| LOC_Os07g42400.1 | OsGATA23a | ACACTCCTGTTTCTAATATG | GTATCGTGGAGCAGGCACCG |
| LOC_Os07g42400.2 | OsGATA23b | TATGTGGCAAATGGAAATGGT | TATTAGAAACAGGAGTGTCT |

**Table S3: Genevestigator data showing the relative expression of various GATA members at different developmental stages of rice as retrieved from** https://www.genevestigator.com/gv/).
